# Cortical distance unifies the extent of parafoveal contour interactions

**DOI:** 10.1167/jov.22.2.15

**Published:** 2022-02-23

**Authors:** Daniel R. Coates, Xiaoyun Jiang, Dennis M. Levi, Ramkumar Sabesan

**Affiliations:** 1 College of Optometry, University of Houston, Houston, TX, USA; 2 Department of Ophthalmology, University of Washington School of Medicine, Seattle, WA, USA; 3 Herbert Wertheim School of Optometry & Vision Science, Helen Wills Neuroscience Institute, University of California, Berkeley, Berkeley, CA, USA; 4 Department of Ophthalmology, University of Washington School of Medicine, Seattle, WA, USA

**Keywords:** contour interaction, cortical magnification, crowding, parafoveal, adaptive optics

## Abstract

It is well known that crowding, the disruptive influence of flanking items on identification of targets, is the primary limiting factor to object identification in the periphery, while limits in the fovea are more determined by the ability to resolve individual items. Whether this is a dichotomous or merely a quantitative difference, and the transition between these two regimes, has remained unexplained. Here, using an adaptive optics system for optimal control of optical and stimulus factors, we measured threshold acuity for identification of Tumbling Es flanked by bars at a variety of flanker spacings and eight eccentricities in the parafovea. Thresholds at each eccentricity were influenced by resolution, contour interaction, and a saturating pedestal effect. When target-flanker spacing was plotted in terms of cortical distance, a single canonical clipped-line fit unified the resultant curves. The critical spacing for letters flanked by bars was found to be 1.3 to 1.5 cortical millimeters, corresponding to approximately 0.1*E outside the fovea.

## Introduction

What limits the ability to identify a letter? In the periphery, object recognition is most limited by crowding—the detrimental influence of flanking contours on target identification (Levi, 2008; Pelli, 2008). In the fovea, identification ability is more constrained by the size needed to resolve individual items, which is determined by sampling resolution and optical blur (Coates et al., 2018; Song et al., 2014). Is this relationship a dichotomous, qualitative difference or merely a quantitative one, and what is the transition between these two regimes? Here we set out to answer this question by measuring the ability to identify letters in the fovea and parafovea flanked by bars, which are known to cause contour interaction, a contributor to crowding (Flom, 1991). While Flom (1991) proposed that the crowding effect is more general, including eye movements and attention as well as contour interaction, here we simply define contour interaction as crowding with bars instead of crowding with letters. To precisely control stimulus characteristics and optical quality with high fidelity, we employed an adaptive optics (AO) system. We measured flanked acuity at an extensive range of eccentricities and flanker spacings to identify the gradient of contour interactions for targets located in the fovea and parafovea.

While similar measurements have been performed previously without AO (Jacobs, 1979), there has been less effort to precisely determine the so-called *critical spacing*, or the spatial extent of the interference from surrounding bars. The critical spacing with letter flankers, on the other hand, has been better characterized across the visual field (Bouma, 1970; Toet & Levi, 1992). It has been known for some time that the critical spacing for crowding is small in the fovea and large in the periphery, but formulations that try to reconcile these findings can be problematic. For example, “Bouma's rule” (the critical spacing divided by the eccentricity is approximately 0.5) yields mathematically problematic effects in the fovea, where the value becomes infinite (Strasburger et al., 2011). Whether flankers affect targets differently in the fovea versus periphery has remained a matter of important debate. Levi et al. (2002) and Song et al. (2014) found that in the fovea, critical spacing scales with target size (unlike in the periphery), while Hess et al. (2000) proposed that foveal vision is limited by optics (rather than neural influences) on the basis of contrast polarity effects.

Recently, several studies have confirmed that the spatial extent of crowding with bars is significantly smaller than that of crowding with letters (Musilová et al., 2018; Marten-Ellis & Bedell, 2021). Here we extend these studies by determining a specific formulation for the spatial extent of crowding from bars. To anticipate our results, we found that bar-flanked acuity threshold versus flanker spacing curves had a complex shape, in agreement with Jacobs (1979), suggesting the involvement of multiple underlying mechanisms. The curves had three distinct zones: a resolution-limited portion (unaffected by distant flankers), a portion showing contour interaction with intermediately spaced flankers, and a saturating portion with proximal flankers that we propose is due to a pedestal-like contrast effect. The varied curves collapse to a single template when expressed in terms of threshold elevation versus *cortical extent*, in line with the results of Levi et al. (1985), who found a similar unifying relationship to characterize the extent of interference from tiny flanking bars on a vernier task.

## Method

An AO microstimulator described previously (Jiang et al., 2019) was used to image the retina and deliver stimuli. Briefly, the instrument consists of an AO scanning laser ophthalmoscope (AOSLO) that imaged the retina with 840 ± 15 nm wavelength. In conjunction, a 543 ± 15 nm wavelength derived from a supercontinuum source provided the stimulus with the help of an acousto-optic modulator (AOM). Both wavelengths were raster-scanned (512 × 512 pixels) on the retina to generate an imaging field. The stimuli were shown as a decrement by modulating the AOM to the OFF position, thereby removing light at the corresponding pixel coordinates. The stimuli appeared to the subjects as a dark target on a 543-nm pseudo-monochromatic background. For all experiments, the optical aberrations of the eye were measured using a Shack–Hartmann wavefront sensor operating at 900 ± 16 nm and corrected with a deformable mirror in closed-loop operation; correction was performed continuously during each trial, although the image was not stabilized on the retina. Monochromatic viewing along with AO correction alleviated blur in the stimulus caused due to the optics of the eye. Psychophysics was performed where the images of cone photoreceptors appeared best focused in the 840-nm wavelength. Subjective image quality was made equivalent for 840 nm and 543 nm, and then only 840-nm cone images were used for assessing the stimulus focus.

In typical operation of the instrument, the imaging and stimulus field subtended up to 1.25 deg. However, at greater eccentricities with lower performance, a larger field was essential to enable larger stimuli and wider flanker spacings. An extra achromatic lens-based telescope was added to magnify the field of view to 2 to 2.4 deg for experiments performed at eccentricity equal to and greater than 2.5 deg. This telescope could be easily introduced and removed into the light path using a flip-in mirror such that both the small and large fields of view were readily accessible. For the smaller field (used at 2 deg or less eccentricity), each raster pixel subtended approximately 0.15 arcmin, while for the larger field, pixels were approximately twice as large.

**Figure 1.**
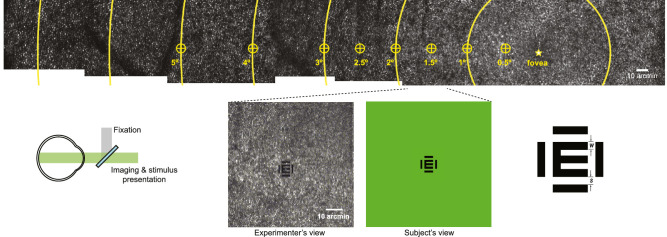
Experimental methods. An AO retinal image (top) is shown where yellow rings represent 1 deg nasal to 6 deg temporal eccentricity. Performance was measured at the indicated locations ranging from the fovea to 5 deg temporal. The eccentricity was varied by an external fixation target while imaging and stimulus presentation were conducted in an AOSLO (bottom left). A representative AO retinal image frame at 1 deg temporal eccentricity showing the experimenter's and subject's view (bottom middle). The experimenter observes the structure of individual cone photoreceptors and the flanked E stimulus rendered into the image frame. The subject views a 543-nm wavelength square field with the same stimulus. The stimulus configuration is shown (bottom right) where an illiterate E is flanked on all sides by bars of the same width. The measures “w” and “s” represent the width of the bars comprising the target and flankers, as well as the nominal edge-to-edge spacing between the target and flanker, respectively.

Stimuli were displayed using a custom software interface developed in MATLAB (MathWorks, Natick MA, USA) described previously (Tuten et al., 2012). Flanked Tumbling E visual acuity was measured using a self-paced four-alternative forced-choice paradigm, in which the stimulus size was varied in a 30-trial QUEST staircase, with each trial displayed for 150 ms. In each experimental run, acuity was measured with flanking bars (Figure 1) at a nominal edge-to-edge spacing of 1, 2, 3, 4, or 5 bar width or in an unflanked condition. At least three repeat measurements were obtained at each nominal spacing and eccentricity. Eccentricity was varied (0, 0.5, 1, 1.5, 2, 2.5, 3, 4, 5 deg temporal retina) using a fixation target provided via an external projector coupled into the AOSLO via a beamsplitter.

Two cyclopleged (0.5% tropicamide) subjects free of retinal disease participated in the study. Both were experienced psychophysical observers. The research was approved by the University of Washington institutional review board, and all subjects signed an informed consent before their participation and after the nature and possible consequences of the study were explained. All procedures involving human subjects were in accordance with the tenets of the Declaration of Helsinki.

### Psychophysical procedure

While classic studies have utilized flanked acuity to measure interference effects previously (Jacobs, 1979; Latham & Whitaker, 1996), the method has seen less use in crowding research until recently (Gurnsey et al., 2011; Coates et al., 2013; Chung, 2014; Song et al., 2014). The more common approach nowadays is to test target and flankers of a single size at different spacings and construct a performance versus spacing curve (Toet & Levi, 1992). The two methods give comparable results for critical spacing, with empirical and theoretical issues summarized in Coates et al. (2021).

In the flanked acuity paradigm, flanker spacing is defined by nominal units, which are multiples of the target letter size. Within a single run (often a staircase procedure), the entire stimulus (both target and flankers) is scaled, and thus both the target and flanker letter size, as well as the absolute flanker spacing (in degrees of visual angle), are modulated. This procedure has several advantages. First, it minimizes trials in that it does not require a separate run to determine threshold sizes at each eccentricity. By scaling sizes, differing conditions are normalized experimentally to equivalent performance levels. Theoretically, results from just an unflanked measurement and a single nominal spacing may be sufficient to determine the critical spacing (Song et al., 2014), although this depends on assumptions about testing conditions. Critical spacings obtained with this procedure have been shown to be consistent with critical spacings obtained with the more commonplace procedure of fixing the letter size and testing different absolute flanker spacings (Coates et al., 2021). On the other hand, this procedure is sensitive to limitations from both target resolution as well as influence from the flankers, so the source of errors in a given condition may not be readily apparent, although we discuss an analysis that addresses this issue.

## Results

**Figure 2.**
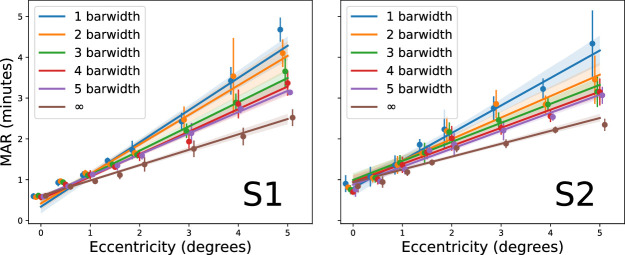
Flanked acuity as a function of eccentricity. Each color indicates a different nominal spacing (in bar widths as shown by the legend), with unflanked targets represented by “infinity.” Error bars show standard deviation of three to four repeated threshold measurements.

The flanked acuity procedure yields threshold target sizes at different *nominal* flanker spacings. Figure 2 presents one way to plot the results, where lines connect the points between each of the nominal spacings tested (1–5 bar widths and unflanked), with eccentricity on the x-axis. This plot clearly shows the deleterious effect of the flanking bars on flanked acuity as a function of eccentricity. The lowest curve in each panel shows the unflanked acuity, with each nominal flanker spacing above indicating steeper curves. This steepness indicates an increasing influence from flankers at further eccentricities. The most strongly flanked stimulus (1 bar width) at 5 deg had a threshold nearly twice the unflanked threshold. Since the entire stimulus (including target, spacing gap, and flanking bars) is expanded/contracted in the procedure, the basis of this paradigm is that thresholds increase in order to push the flankers outside the interference zone, which extends radially or elliptically from the center of the target letter (Toet & Levi, 1992; Song et al., 2014).

**Figure 3.**
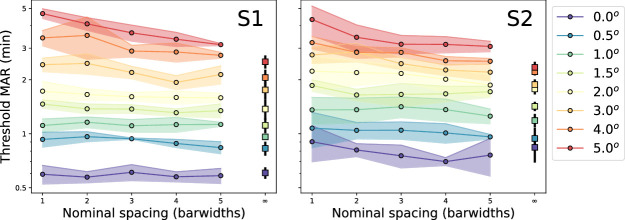
Acuity thresholds as a function of nominal flanker spacing, for each eccentricity (indicated by color; see legend). Squares indicate unflanked trials. Shaded regions and error bars indicate ± 1 standard deviation of repeated measurements.

An alternative way to plot data is shown in Figure 3, where the eccentricities are stacked, with threshold plotted versus nominal flanker spacing. Several trends are revealed with this presentation. Toward the fovea (cool colors, bottom-most curves), there is little impact of flankers on target recognition, leading to primarily flat curves. On the other hand, at larger eccentricities (hotter colors), flankers exert more of an influence; at 5 deg, performance worsens with flanker proximity. At the furthest spacing tested (5 bar widths, rightmost red circle), the threshold is different from the unflanked threshold (red square). The intervening eccentricities show a mixed pattern: at 1 to 3 deg, curves have a relatively flat portion for the most proximal flankers, then a gradual or abrupt fall-off to the unflanked threshold beyond some critical spacing. A nearly identical pattern was observed previously with Landolt Cs flanked by bars at similar flanker widths and eccentricities (Jacobs, 1979).

Next, since it is known that crowding effects are best understood as size-independent interactions around a zone centered on the target letters (Toet & Levi, 1992; Song et al., 2014), we converted from nominal spacing to absolute units of visual angle, in terms of the center-to-center spacing between the target letters and the flanking bars. For example, flanking bars 2 bar widths from the target will have their center exactly one letter size away from the center of the target. Mathematically, the conversion used is: absolute center-to-center spacing = letter_size * (bar widths + 3) / 5. The 3 occurs since exactly 3 bar widths separate the center of a target E and the center of an abutting flanking bar. The 5 divides the letter size into bar widths based on the Sloan letter proportions.

**Figure 4.**
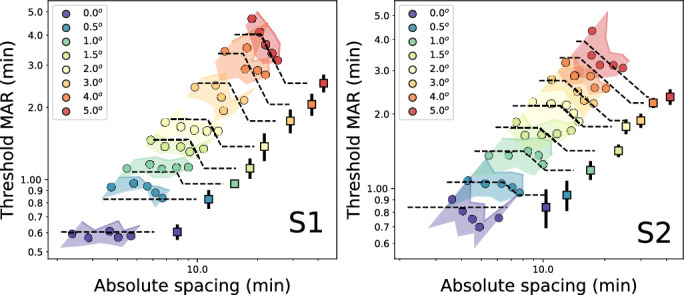
Data from Figure 3, replotted with absolute center-to-center spacing on the abscissa. Solid colored regions indicate ± 1 standard deviation from each point, projecting diagonally due to conversion to absolute spacing. Dashed curves show clipped line cortical spacing fits. Points with black center denote points used for crowding portion of fit (see text for details).

The results of this conversion are shown in Figure 4, which are comparable to the families of curves seen previously for other types of flankers (Latham & Whitaker, 1996; Gurnsey et al., 2011; Song et al., 2014). As in Figure 3, eccentricities are naturally arrayed from the bottom (fovea: lower thresholds) to the top (larger eccentricities: higher thresholds). At larger eccentricities, the curves begin to tilt vertically, again revealing an increased influence of flankers. In our case, the steepest slope seen was approximately −0.85 on a log-log scale, rather than the −1 slope observed with more complex flankers (Gurnsey et al., 2011; Song et al., 2014).

The points in Figure 4 are less regular than those reported in the crowding studies; intermediate eccentricities have asymptotic flat portions at the closest spacings. Therefore, to model the curves at each eccentricity, we used a clipped line curve (dashed lines in Figure 4) with a flat portion at the closest spacings, a negatively sloped portion at intermediate spacings, and a flat portion at more distant flanker spacings.

To fit these data requires vertical and horizontal shifts to align curves at each eccentricity to a single template function, a procedure that has been performed previously with similar data. Latham and Whitaker (1996) and Gurnsey et al. (2011) both divided the ordinate by a factor that captures the expected linear change in unflanked thresholds with eccentricity. Latham and Whitaker (1996) used a similar shift for the abscissa (with a much smaller coefficient), while Gurnsey et al. (2011) found that raising the eccentricity to a power greater than 1 was necessary to sufficiently align their curves. Song et al. (2014) divided the spacing by the eccentricity plus a small number (0.45).

Here we took a different approach that was anatomically inspired. We retained the shift in ordinate corresponding to flanker elevation (from unflanked size at the corresponding eccentricity) but expressed the abscissa shift in terms of *cortical separation* —presumed distances between stimuli in the primary visual cortex; a transformation utilized previously to characterize interaction extents for Ts flanked by Ts (Tripathy & Levi, 1994) and verniers flanked by small bars (Levi et al., 1985).

The predominant expression for cortical distance that has been proposed is the conformal mapping (Schwartz, 1977), which states that the cortical distance from the fovea is proportional to the complex logarithm of the eccentricity. To overcome singularities at the fovea, Schwartz (1980) extended the formula to *k* * log (*z* + *a*) for visual field location *z* (a complex number to capture two dimensions), with parameter *a* typically between 0.6 and 1, and *k* (or $M_{0}^{-1}$), the magnification at the fovea, which is estimated to range between 15 and 25 mm/deg for humans. Since all of our targets were presented along the horizontal meridian, the simple *k* * log(*E* + *a*) can be used for eccentricity *E*. Cortical distance for a visual angle θ at eccentricity *E* is determined from the equation dist(E+θ)-dist(E), simplifying to $\log(\frac{E+a+\theta }{E+a})$. More exhaustive descriptions and comparisons of different cortical magnification functions are given in Strasburger et al. (2011) and Hussain et al. (2015). In the absence of anatomical data for our subjects, we used standard estimates from the literature for human cortical magnification from functional MRI (Duncan & Boynton, 2003; Larsson & Heeger, 2006) to determine parameters that resulted in optimal curve alignment.

**Figure 5.**
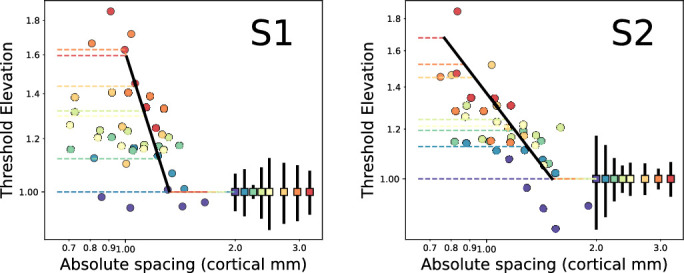
Same data points and fits from Figure 4, collapsed based on cortical extent (abscissa) and threshold elevation from unflanked targets (ordinate). Clipped lines describe the data at each eccentricity well, with a sloped crowding portion (thick black line), unflanked performance beyond the critical spacing (lower right of graph, at *y* = 1), and asymptotic values for close spacings, specific to each eccentricity (colored lines).

Figure 5 shows the data collapsed in terms of the absolute spacing (in cortical distance in millimeters) on the x-axis and threshold elevation on the y-axis. Threshold elevation is calculated relative to the mean of the unflanked trials. A clipped line for each eccentricity has three regions: proximal saturated threshold (which varied across eccentricities; colored lines); the asymptotic floor performance, right side of curve, by definition always 1; and a negatively sloped portion. To determine the optimal coefficients (independently for each subject), we performed a grid search to minimize the mean squared error (of log thresholds) over four parameters: the cortical “a” term, the slope of the flanker interference line, the critical spacing (beyond which there is no flanker effect), and the critical spacing for saturation with near flankers. Preliminary analysis revealed that the flanker critical spacing was a fixed value in terms of cortical spacing, while the saturation critical spacing could be expressed as a multiple of the corresponding unflanked bar size threshold at each eccentricity. The range of values tested revealed a single minimum for each subject, despite certain regions of the curve being underconstrained. For example, there are few data points on the sloping portion for S1, likely making the slope artificially steep and the critical spacing smaller. The resultant best parameters and search ranges are shown in Table 1.

**Table 1.** Parameter fits and grid search ranges. MAR = minimum angle of resolution.

Parameter	Search range	S1 best	S2 best
Cortical constant **“a”**	(0.4, 1.0)	0.78	0.87
Slope	(−3.0, −0.5)	−1.71	−0.76
Critical spacing (mm)	(1.2, 1.9)	1.32	1.51
Saturation spacing (×MAR)	(6, 9)	8.2	6.8

While the coefficient *a* was empirically determined from the results, $M_{0}^{-1}$, or the actual magnitude of cortical magnification, is unconstrained. Larsson and Heeger (2006) used the formula $d=\frac{\log(Ecc)}{\beta }$ to describe distances relative to 3 deg (meaningful only between 1 and 5 deg), with β=0.0577, which corresponds to a foveal magnification of approximately 18 mm/deg. Alternatively, Duncan and Boynton (2003) described cortical magnification with the power function $9.81*Ecc^{-0.83}$ for their data, which was collected between 1.5 and 12 deg. Strasburger et al. (2011) refit their data and estimated $M_{0}$ to be 22.5 mm/deg. Our extrapolated curves lie between these two curves beyond an eccentricity of 3 deg, and are flatter near the fovea.

**Figure 6.**
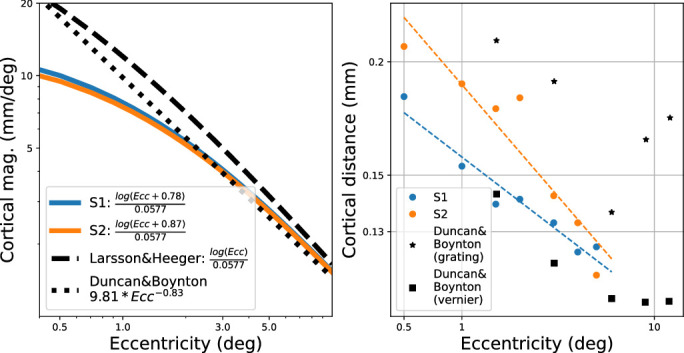
Estimated cortical distances are compared with several results from the literature. Left panel shows cortical magnification curves, while right panel plots unflanked results from our study along with two resolution tasks from Duncan and Boynton (2003).

Figure 6 (left panel) shows the inferred cortical magnification curves for each subject, in relation to these two results from the literature. From 2 to 10 deg, they are nearly indistinguishable. The estimated cortical distance for unflanked targets is shown in the right panel of this plot, along with the estimates from two resolution tasks from Duncan and Boynton (2003). Note the agreement with the shapes of the curves, especially between 1 and 5 deg, where the studies empirically overlap. Further details about the significance of this comparison are given in the Discussion.

A remarkable feature of Figure 5 is the observation that thresholds reduce to unflanked levels when flankers are 1.3 to 1.5 to mm of cortical distance from the center of the target letter, which is an intermediate value between the 1-mm critical cortical separation for small bars flanking vernier targets (Levi et al., 1985) and 5 to 6 mm describing the spatial extent of interaction for Ts flanked by Ts (Tripathy & Levi, 1994). The saturation portions resisted a single spacing constant but instead could be expressed as a multiple (7–8) of the unflanked bar size at each eccentriciy: from 5 to 6 min at half a degree eccentricity to approximately 20 min at 5 deg. The consistency of the canonical function across eccentricities permits the precise determination of the critical spacing for interference from bars, which will be described in detail in the Discussion.

### 180-deg flip errors

One aspect of the psychophysical method employed is that it cannot differentiate errors due to flankers from errors due to resolution (i.e., insufficient letter size). Threshold crowded letter sizes are determined by three influences: the size of the target letter (which may be limited by blur) and the influence of the flankers, which may cause either overlap masking or crowding (Song et al., 2014). However, it has been shown that particular types of error patterns arise due to interactions with flankers. Specifically, Levi et al. (2002) showed that errors for identifying flanked illiterate Es were more likely to be 180-deg flip confusions when presented in the crowded periphery versus more randomly distributed errors in the fovea. We analyzed the present data to determine whether Es flanked by bars also showed characteristic error patterns.

**Figure 7.**
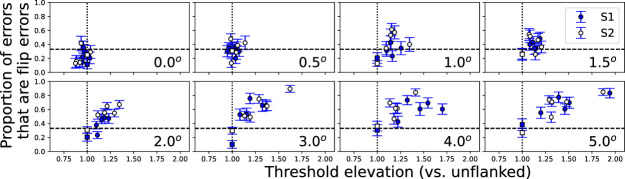
For each eccentricity (indicated in lower right corner), the proportion of errors that are due to flip errors are plotted for each nominal spacing, with error bars indicating the standard deviation across repeats. The abscissa shows threshold elevation from unflanked acuity (i.e., the ordinate of Figure 5). The dashed line indicates a proportion of 1/3, which corresponds to chance level for a flip error. The dotted lines indicate equivalence to unflanked error size. Square symbols indicate unflanked trials.

Figure 7 plots the proportion of errors (out of the total errors in each condition) that were 180-deg flip errors, as a function of threshold elevation versus corresponding unflanked acuity at each eccentricity (i.e., the ordinate of Figure 5). The dashed line indicates 33%, which corresponds to a chance occurrence of flip errors (there are three possible erroneous responses, with exactly one constituting a 180-deg flip of the target). The dotted line indicates thresholds equal to the unflanked acuity threshold. Note that at all eccentricities, unflanked targets (abscissa = 1.0) are near the 33% chance line. As thresholds are elevated from unflanked sizes (indicating error sources other than blur), error patterns shift above the 33% line, indicating a preponderance of flip errors. Importantly, all flanked and unflanked conditions contain approximately the same number of errors, which is controlled by a staircase. Thus, like the pattern seen with letter crowding, errors that can be attributed to the flanking bars are associated with increased proportion of flip errors.

## Discussion

The curves that result from measurements of bar-flanked acuity at eccentricities near the fovea appear surprisingly nonhomogeneous, with each curve in Figure 4 having disparate features, unlike the more regular curves with letter flankers (Gurnsey et al., 2011). Nevertheless, the clipped line fit, with the appropriate units of threshold elevation versus cortical distance, was able to capture the principal characteristics of the functions remarkably well. Different eccentricities sampled different portions of the canonical curve. Specifically, the “critical spacing” for contour interaction, beyond which no flanker-specific reduction in performance is observed (right-most flat portion), can be described by a constant term in cortical space. Next, for flankers just inside this critical spacing, a sloped portion of the curve is consistent across eccentricities, constituting a canonical form for contour interaction.

However, at even nearer flanker spacings, performance again flattens, yielding a saturated, asymptotic portion. This region does not align in cortical space but instead appears as the stacked colored lines in Figure 5. However, we found that the outer border of this region occurs at seven to eight times the unflanked threshold bar size at the corresponding eccentricity. Note that this feature of contour interaction is also clearly visible in the results of Jacobs (1979), who measured acuity for Landolt Cs flanked by bars. The source of the saturation is unclear, although we posit that it is due to a qualitatively different mechanism than the sloping portion that corresponds to crowding, and likely at an earlier stage of processing. One piece of evidence is that the sloped line (including the critical spacing) is consistent across all eccentricities in terms of cortical extent. The critical spacing for the asymptotic portion, on the other hand, can be described as a multiple (7–8) of the unflanked threshold letter acuity. The saturation may mirror the facilitation (or upturn) seen in letter crowding with simple flankers that we (Coates & Levi, 2014; Coates et al., 2018) and others (Siderov et al., 2020) have described in depth. Subjective reports indicated that the closest flanking bars interact with the bars comprising the target Es in a way that provided cues, such as a “double bar” that helps limit target possibilities and lead to better recognition than expected. The flankers may act as *pedestals* to improve performance by introducing low-level (contrast) cues that aid in discrimination of the bars of the E (Coates et al., 2018). While a similar phenomenon with elementary tasks such as gap detection flanked by bars has been reported (Takahashi, 1968), it is possible that this effect would be abolished with other types of targets or flankers. However, Siderov et al. (2020) demonstrated the nonmonotonic influence of flanking bars on Sloan letter identification. In the current study, the extent of the saturation zone could be roughly approximated by a constant multiple of the unflanked acuity, suggesting a mechanism more closely tied with resolution acuity, unlike crowding or contour interaction.

We chose to normalize the ordinate of each curve to the corresponding unflanked size threshold at the corresponding eccentricity. In theory, this could be interpreted as normalizing to local units of cortical distance, assuming that threshold unflanked acuity reflects constant cortical scaling (Duncan & Boynton, 2003). On the other hand, it is clear from Figure 6 (right panel) that the estimated cortical distance for acuity targets within 5 deg of the fovea is not constant but rather increases with more central targets, in our results as well as prior literature. Further experiments are needed to precisely characterize the relationship of acuity and cortical distance near the fovea.

Flanked size thresholds are related to traditional clinical measures of visual acuity, (Jacobs, 1979; Latham & Whitaker, 1996; Coates et al., 2013; Chung, 2014; Song et al., 2014) but differ markedly from the majority of studies of crowding and contour interaction (Flom et al., 1963; Toet & Levi, 1992; Kooi et al., 1994; Coates et al., 2018; Marten-Ellis & Bedell, 2021). In these latter studies, an appropriate target size is chosen and target-flanker spacing (but not target size) is varied. Due to the demands of AO psychophysics, it is desirable to limit the duration of testing and make optimal use of each trial spent in the system, motivating the use of adaptive procedures such as QUEST. With the flanked acuity paradigm, there is no need to pretest to determine unflanked performance at each eccentricity. It has been observed that sampling unflanked acuity and only one nominal spacing may be sufficient to define an entire set of results (Song et al., 2014; Coates et al., 2013). However, this technique rests heavily on assumptions about the shape of the underlying functions, which has been well characterized for crowding with letters in the periphery (Song et al., 2014; Coates et al., 2013). Our characterization of the resultant functions now permits the use of an optimized procedure for bar flankers.

One aspect of the flanked acuity paradigm is that errors can be due to either interference from the flankers or from limitations due to insufficient spatial resolution (Song et al., 2014). Isolated measurements cannot differentiate between these two influences, although when thresholds are larger than the unflanked size threshold, errors other than blur (e.g., from the flankers) are suggested. Figure 6 shows that the proportion of flip errors observed differs between these two sources of error, with significantly greater proportion of flip errors as thresholds are elevated from unflanked sizes, despite the same overall performance level in each case. Thus, it would be possible for a psychophysical procedure to use this information to determine error sources, which may be necessary for an adaptive testing procedure. Note, however, that it is unclear if similar effects would be observed with other targets or with other types of flankers. Dakin et al. (2010) found few 180-deg errors (20%) for Ts flanked by a single flanker composed of a vertical and horizontal line.

Cortical scaling has been employed before to describe the interference on vernier acuity of flanking bars at several locations in the visual field (Levi et al., 1985), finding an extent of approximately 1 mm. Tripathy and Levi (1994) expressed their measures of the extent of interactions for T targets and T flankers in terms of cortical distance, estimating 5 to 6 mm, by scaling up estimates from monkeys. More recently, Pelli (2008) has pointed out how the rule-of-thumb estimate of the critical spacing for crowding of approximately half the eccentricity (Bouma, 1970) corresponds to about 6 mm on the visual cortex, using the same estimate of Larsson and Heeger (2006) that we utilized. Mareschal et al. (2010) found that the influence of Gabor flankers on a Gabor target switched between attraction and repulsion at approximately 0.5 mm.

**Figure 8.**
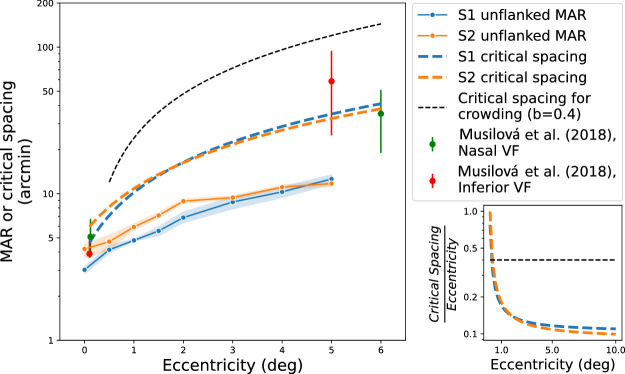
Summary of minimum angle of resolution (MAR) and critical spacing for bar and letter flankers. Empirical unflanked resolution is shown by solid points and lines (shaded region indicates standard deviation of thresholds). Fitted critical spacings from the cortical extent model are shown by colored dashed lines. Red and green points show empirical critical spacings for Sloan letters and bar flankers from another study (Musilová et al., 2018). Black dashed curve indicates crowding with letters, with stereotypical critical spacing of 0.4*E. Inset shows estimated ratios of critical spacing divided by eccentricity, for stereotypical letter flankers and from our model of bar flankers.

The efficacy of the cortical scaling model allows the estimation of the exact extent of the interference from the flanking bars. The estimates of 1.3 mm for S1 and 1.5 mm for S2 would correspond to points on crowded proportion correct versus flanker spacing psychometric functions near the unflanked asymptote. Musilová et al. (2018) estimated contour interaction at various luminances and eccentricities and characterized critical spacing as the distance at which performance drops by $\frac{1}{e^{3}}$, which is a conservative definition of the critical spacing, comparable to our use of the intersection with the asymptotic line. Figure 8 plots our estimates, along with the empirical measurements reported by Musilová et al. (2018). Note the two colors indicate different sets of observers and different visual field locations. Lateral interactions in the inferior visual field (red points) would be expected to have a larger extent than on the horizontal meridian (Greenwood et al., 2017) where we tested. Edge-to-edge distances have been converted to center-to-center distances by adding three fifths of the target letter size as described above.

To contrast our results to crowding with letters, we have plotted the function representing a Bouma ratio ($\frac{Critical\;spacing}{Eccentricity}$) of 0.4 (which corresponds to a critical spacing of around 6 mm). The plots of the “Bouma ratios” (ratio of critical spacing to eccentricity) for our results of flanking with bars are shown in the inset. The functions of both observers asymptote near 0.1 for eccentricities greater than 2 deg, rising sharply near the fovea, as predicted by Strasburger (2020). It has been known for some time that bars cause less interference than letters (Flom, 1991) and has been recently demonstrated directly (Marten-Ellis & Bedell, 2021). Possible reasons for the difference include the inability to confuse a target and flanker, asimilarity between letter targets and bar flankers (Kooi et al., 1994; Bernard & Chung, 2011), and the lack of complexity of the bar flankers (Bernard & Chung, 2011). Flom (1991) proposed that the more general phenomenon of crowding includes the interference effects from nearby contours (contour interaction), as well as impacts from eye movements and the effects of attention. We believe that the evidence supporting qualitatively different effects in the fovea may reflect experimental limitations, such as inadequate optical correction, which we have overcome with AO. The scale of foveal flanker effects is so small (Coates et al., 2018) that experimental manipulations to reduce visibility such as lowering the contrast (Song et al., 2014) or blurring (Song et al., 2014) may reveal the paradigm-conflated limitations of resolution or overlap masking, rather than flanker interference.

In summary, we have shown that despite the varied nature of results for bar-flanked letter acuity in the fovea and parafovea, expressing results in terms of cortical distance standardizes the curves across eccentricities. The critical distance of 1.3 to 1.5 cortical millimeters (asymptoting at approximately 0.1*E for larger eccentricities) may correspond to the size of canonical cortical processing modules (Levi et al., 1985) or to sampling by a fixed number of retinal ganglion cells (Kwon & Liu, 2019). While some aspects of our results may be unique to bar flankers (such as the asymptotic zone with proximal bars), differences between crowding with bars and crowding with letters may simply be quantitative, captured by the coefficients of the crowding region of the canonical curve. Further, the ability of the template clipped line to describe the results suggests that common mechanisms underlie flanked acuity across the visual field, including near the fovea.
